# Effect of Two-Stage Cooling on the Microstructure and Tribological Properties of Steel–Copper Bimetals

**DOI:** 10.3390/ma15020492

**Published:** 2022-01-10

**Authors:** Yuanyuan Kang, Guowei Zhang, Zhaojie Wang, Hong Xu, An Wan

**Affiliations:** 1 School of Material Science and Engineering, North University of China, Taiyuan 030051, China; kangyy469627632@163.com (Y.K.); 20030358@nuc.edu.cn (G.Z.); wzj1997vip@163.com (Z.W.); 2 Beijing North Hengli Technology Development Co., Ltd., Beijing 100070, China; wanna93@163.com

**Keywords:** EN CC497K, steel–copper bimetal, solidifies, friction and wear

## Abstract

In this paper, the solid–liquid composite method is used to prepare the steel–copper bimetal sample through two-stage cooling process (forced air cooling and oil cooling). The relationship between the different microstructures and friction properties of the bimetal copper layer is clarified. The results show that: the friction and wear parameters are 250 N, the speed is 1500 r/min (3.86 m/s), the friction coefficient fluctuates in the range of 0.06–0.1, and the lowest point is 0.06 at 700 °C. The microstructure of the copper layer was α-Cu, δ, Cu_3_P, and Pb phases, and Pb was free between α-Cu dendrites. When the solidification temperature is 900 °C, the secondary dendrite of α-Cu develops. With the decrease temperature, the growth of primary and secondary dendrites gradually tends to balance at 700 °C. During the wear process, Pb forms a self-lubricating film uniformly distributed on the surface of α-Cu, and the Cu_3_P and δ phases are distributed in the wear mark to increase α-Cu wear resistance.

## 1. Introduction

Bimetallic materials are composed of two or more metals with different properties that are combined in a layered manner to form a metallurgical interface [1,2,3]. Steel–copper bimetallic materials are widely used in the axial piston pump [4,5], and copper-based alloys, as one component of the bimetal, play an unreplaceable role due to their outstanding self-lubricating properties [6,7]. The EN CC497K alloy exhibits excellent tribological properties due to the high Pb content [8,9,10]. Therefore, in this paper, we select EN CC497K as the research object of the bimetallic copper layer.

The steel–copper bimetal bonding methods include: solid–liquid casting, electron beam welding, explosive welding, semi-solid rolling, selective laser melting, etc. [11,12,13,14]. The microstructure of the copper layer produced by traditional casting processes show a coarse reticular dendritic structure, which leads to intergranular segregation and negative segregation, and hot-cracking in metal materials [15]; all of these defects severely restrict the tribological performances of materials. Numerous studies have reported that incorporating additives, such as graphite, molybdenum disulfide, titanium diboride, nickel, and silver powder, into the alloy matrix can improve the anti-wear and mechanical properties of the lubricating alloy [16,17,18,19,20,21]. However, the structure of the bimetallic copper layer formed at room temperature is still coarse, the hardness of the copper layer is low, and the wear resistance is insufficient.

The purpose of this work is to accelerate the solidification cooling rate during solid–liquid forming by forced air cooling, refine the α-Cu dendritic, and preserve the copper layer structure at different solidification temperatures through oil quenching. The microstructures under different solidification temperatures are discussed, and the lubrication mechanism of copper with different microstructure morphologies is proposed in combination with friction and wear properties.

## 2. Experimental Procedure

### 2.1. Material and Characterizations

Using the solid–liquid casting method to prepare steel–copper bimetal samples, the preheating temperature of the steel matrix was 1150 ± 30 °C and the copper alloy smelting temperature was 1200 ± 20 °C. The copper alloy was subsequently poured into the preheated steel matrix to form a bimetallic composite interface.The composition of the steel after casting is 0.42% C, 0.21% Si, 0.68% Mn, 0.04% Cr, and 0.02% Ni (wt.%). The grade of copper alloy is EN CC497K, and the actual element content obtained is shown in Table 1, which were detected by the German SPECTRO-MAXx spectrometer.

**Figure 1:**
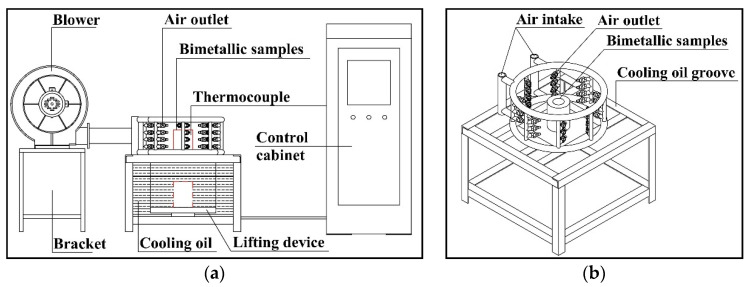
Steel–copper bimetal two-stage cooling device. (**a**) Device control module; (**b**) the steel–copper bimetal placement.

Figure 1 is a schematic diagram of the steel–copper bimetal two-stage cooling device. After the solid–liquid forming, the bimetal is placed in the position of the red solid line in Figure 1a for forced air cooling. The copper liquid cooling temperature is monitored by a temperature sensor: when the solidification temperature reaches the preset temperature (400–900 °C), the bimetal sample is lowered to the position of the red dotted line, as through cooling the oil rapidly solidification occurs to obtain bimetal samples.

**Figure 2:**
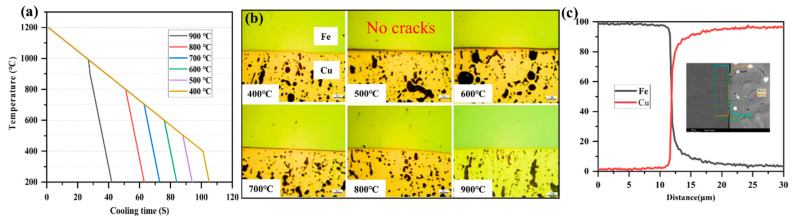
The bimetal rapid solidification test parameters and steel-copper interface. (**a**) Test parameters of the steel–copper bimetal; (**b**) OM microstructure; and (**c**) Fe–Cu atomic interdiffusion.

The aim of the experiments was to identify a solidification temperature that avoids cracking between the steel and copper interface, with a desirable microstructure to optimize friction and wear properties. Solidification temperatures of 400 °C, 500 °C, 600 °C, 700 °C, 800 °C, and 900 °C were investigated. Figure 2 shows the microstructure of the steel–copper bimetal interface at different solidification temperatures: the bimetal copper layer is composed of α-Cu and Pb, and Pb and Cu are not mutually soluble at all, being free on the Cu matrix. With the decrease in the solidification temperature, Pb changes to varying degrees, and the tribological properties of the copper layer also change. The steel–copper interface prepared formed a metallurgical bond without defects, as shown in Figure 2c.

### 2.2. Test Method

**Figure 3:**
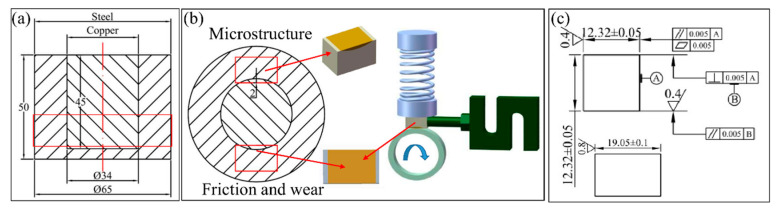
Bimetal samples size and sampling method. (**a**) Bimetal size before sampling; (**b**) microstructure and friction sampling location; and (**c**) size of the samples.

The samples size and sampling method are shown in Figure 3. In the actual working conditions of the plunger pump cylinder, the friction surface of the plunger hole copper layer was 2 mm from the interface thickness, so the friction and wear copper layer was taken as 2 mm during the sampling process. Two samples were taken from symmetrical positions, and they were used for microstructure and friction tests.

According to the specification of GB/T 12444-2006, the friction and wear test was carried out on an MRH-3 high-speed ring-on-block wear tester. The testing of the oil-rich working conditions was performed in a vehicle general lubricating oil of 15W-40. The material of the friction counter ring was 534A99, and its surface roughness was Ra = 0.6, with the copper layers of the samples having the same surface roughness. The specific working conditions and methods used in this article were: the test load was 250 N, the speed was 1500 r/min (3.86 m/s), and the friction time was 20 min. The wear test is shown schematically in Figure 3b; in the oil-rich condition, lubricating oil continuously covered the contact surface of the friction pair during the test.

The friction coefficient was automatically acquired and recorded by the tester, the wear volume (V, mm^3^) was measured by a 2D surface profiler, and the wear rate was cal-culate by the following equation:

Wear rate = V/(F·L)

where F is the load, and the L is the total distance duringthe friction test.

The hardness test of copper layer was carried out with a Brinell hardness tester, with the test parameters being 2.5 kN load force and the holding time 30 s. The copper alloy was etched with NH_3_·H_2_O:H_2_O_2_ = 1:5, and the steel was etched with a 5% nitric acid alcohol solution. A Zeiss Smartzoom 5 optical microscope (OM) was used to observe of the bimetallic samples. The copper alloy phase and wear scar were studied by field-emission scanning electron microscopy (SEM) operated at 20 kV combined with energy-dispersive spectrometry (EDS).

## 3. Results and Discussion

### 3.1. Friction and Wear Properties

**Figure 4:**
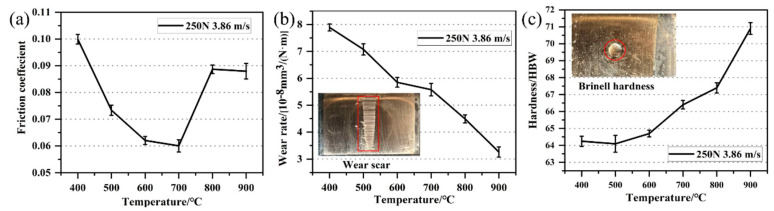
Copper layers properties. (**a**) Friction coefficient; (**b**) wear rate; and (**c**) Brinell hardness.

Figure 4 shows the friction coefficient, wear rate and hardness of copper layers at different solidification temperatures. Figure 4a shows the friction coefficient fluctuates in the range of 0.06–0.1 during the change of the solidification temperature from 400 °C to 900 °C, and it first decreases and then increases as the cooling temperature increases [22]. It reaches the lowest point of 0.06 at 700 °C, and the friction coefficient stabilizes after 800 °C. Figure 4b,c shows that the wear rate and hardness show an opposite trend; the wear rate gradually decreases with the increase in cooling temperature, and the hardness gradually increases.

### 3.2. Microstructure of Copper Layers

**Figure 5:**
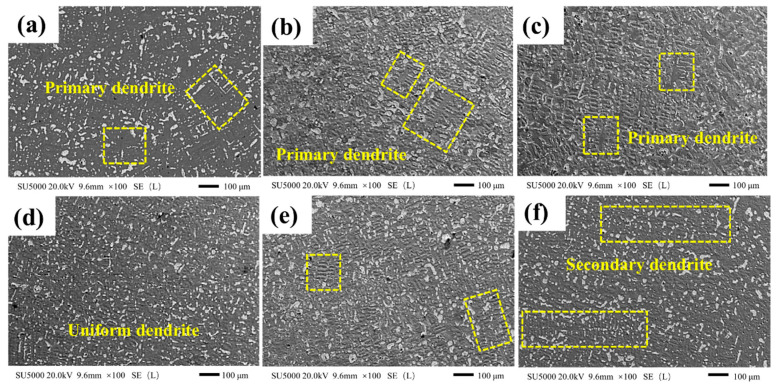
Copper layers dendrite morphology at different solidification temperatures. (**a**) 400 °C, (**b**) 500 °C, (**c**) 600 °C, (**d**) 700 °C, (**e**) 800 °C, and (**f**) 900 °C.

Figure 5 shows the SEM images of copper at different temperatures. It can be seen that, with the gradual decrease in the temperature, the α-Cu dendrite morphology in the alloy changes significantly. At 900 °C, the secondary branches on the same tree trunk of the crystal growth are developed; at 800 °C, the number of secondary dendrites is evidently reduced, mostly of a short and thick morphology; at 700 °C, the structure of the α-Cu matrix forms uniform dendrites, and Pb is distributed among the dendrites; at 600 °C to 400 °C, the primary dendrite arm spacing gradually widens.

The high temperature provides the driving force for the dendrite growth and nucleation [23]. When the solidification temperature is high, the α-Cu is directionally solidified under the rapid cooling, the dendrite growth is thin and long, and the secondary dendrite is developed. With the decrease in temperature, the growth of primary dendrite and secondary dendrite gradually tends to balance, reaching the best at 700 °C, and the dendrite distribution is more uniform. With the gradual extension of the solidification, the primary dendrite arms grow gradually, until the temperature drops to 400 °C, the dendrites interlace each other. During the solidification process, the low melting point Pb is squeezed between the dendrites, and the different α-Cu morphologies result in different Pb morphologies.

**Figure 6:**
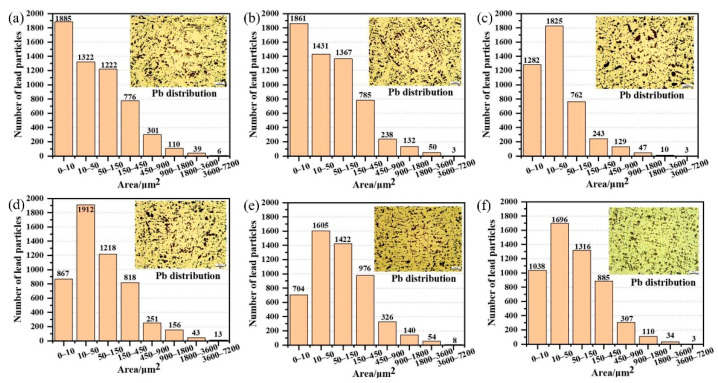
The copper layer Pb statistics. (**a**) 400 °C, (**b**) 500 °C, (**c**) 600 °C, (**d**) 700 °C, (**e**) 800 °C, and (**f**) 900 °C.

Figure 6 is a statistical diagram of the number of Pb in the copper layer. It can be seen that the area of Pb is mostly concentrated within 450 μm^2^, and the uniform and rounded state of Pb is conducive to the wear resistance of copper layer. Comparing the morphology of Pb at different solidification temperatures, the Pb particle size at 400 °C and 500 °C accounts for a larger proportion of 10 μm^2^, and when the temperature exceeds 600 °C, the number of Pb with 10–50 μm^2^ is the largest. Pb has a face-centered cubic structure, and its shear strength is low. During friction, it can effectively reduce the friction coefficient through the self-shearing of lead between the friction pairs. In the friction direction, due to the shear force, it transfers to the surface enrichment and forms a self-lubricating Pb film that improves the friction properties of the alloy.

**Figure 7:**
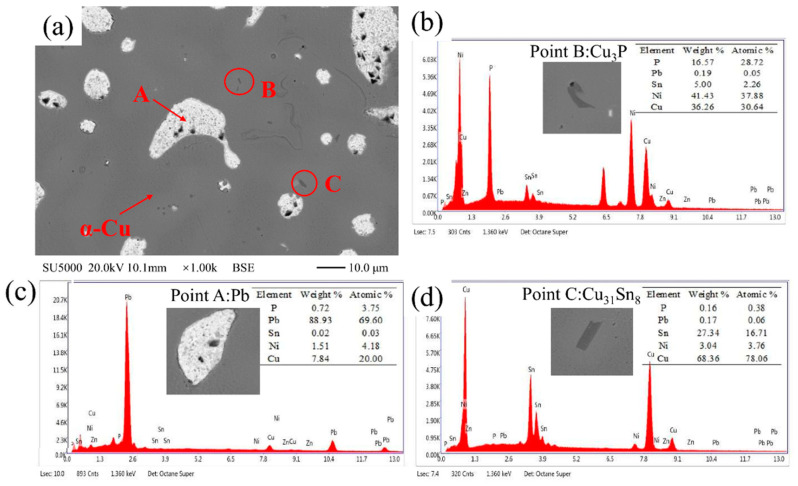
The copper layer phase composition. (**a**) BSE microstructure, (**b**) EDS at point B in (**a**), (**c**) EDS at point A in (**a**), and (**d**) EDS at point C in (**a**).

Figure 7 shows the EDS point scan of each phase in the copper layer. The structure mainly was α-Cu, δ (Cu_31_Sn_8_), Cu_3_P, and Pb phases. The α-Cu matrix is soft, and δ and Cu_3_P are both hard phases, which can increase the surface hardness of the alloy and improve the wear resistance.

**Figure 8:**
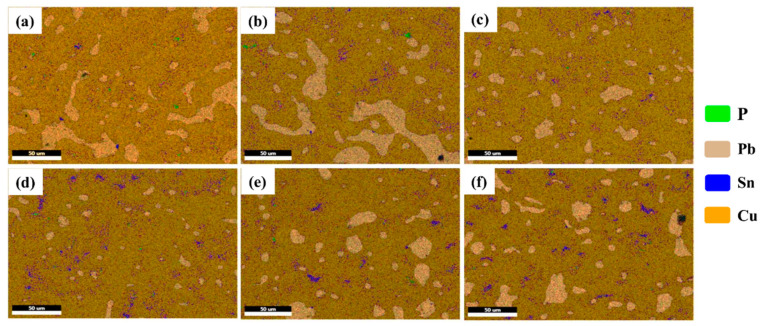
The distribution of elements in the copper layer at different solidification temperatures. (**a**) 400 °C, (**b**) 500 °C, (**c**) 600 °C, (**d**) 700 °C, (**e**) 800 °C, and (**f**) 900 °C.

Figure 8 is the EDS surface scanning energy spectrum at different solidification temperatures. Cu elements are distributed throughout the entire structure, and P and Sn are distributed in the α-Cu matrix, but the Sn content is relatively high above 700 °C. As the solidification progresses, Sn and P elements continue to diffuse into Cu, causing the δ and Cu_3_P phases in α-Cu to change to varying degrees. Sn diffuses into Cu to form (α + δ) peritectic structure [24], making the hard particles in the copper layer reduce, which improves the friction properties. The Cu_3_P phase is a hard phase, which is distributed in the α-Cu matrix and locally enhances the wear resistance of the alloy during the wear process.

### 3.3. Analysis of Wear Mechanism of Copper Layer

**Figure 9:**
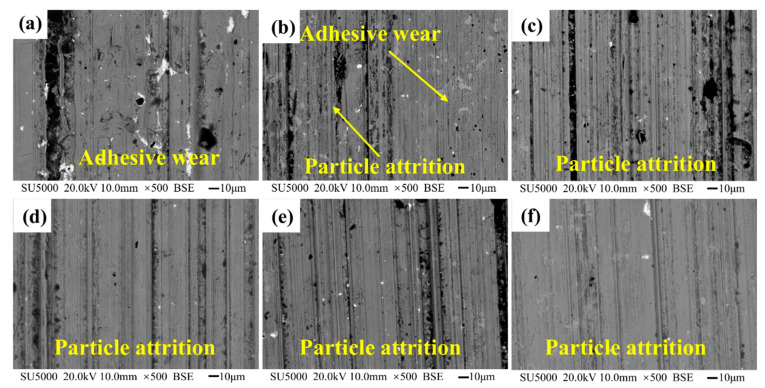
The copper layer wear scar morphology. (**a**) 400 °C, (**b**) 500 °C, (**c**) 600 °C, (**d**) 700 °C, (**e**) 800 °C, and (**f**) 900 °C.

The wear scar of copper layer is observed in Figure 9. Under the conditions of a friction load of 250 N and a linear velocity of 3.86 m/s, as the solidification temperature decreases, the wear marks on the surface of the copper gradually become deeper. When the temperature is higher than 700 °C, the wear surface is relatively smooth, and there are wear marks and wear debris on the substrate. The contact area between the α-Cu matrix and the steel ring is mostly the contact between the small metal protrusions. The developed secondary dendrites can enhance the wear resistance of the α-Cu matrix, which are not easily damaged during the wear process [25]. When the temperature is 700 °C, the distribution of α-Cu dendrites is uniform, and the soft Pb forms a lubricating film on the surface of the substrate, which effectively reduces the friction coefficient; the wear mechanism above 700 °C is particle wear.

When the temperature is lower than 600 °C, the primary dendrite of α-Cu is thickened, and the Pb film is gradually extruded, causing the friction coefficient to increase and causing adhesive wear. The wear intensifies with the decrease in the solidification temperature, the penetration depth of the micro-protrusions on the friction surface of the steel ring increases, the actual contact area between the friction pairs increases, and the wear is serious, resulting in an increase in the wear rate.

**Figure 10:**
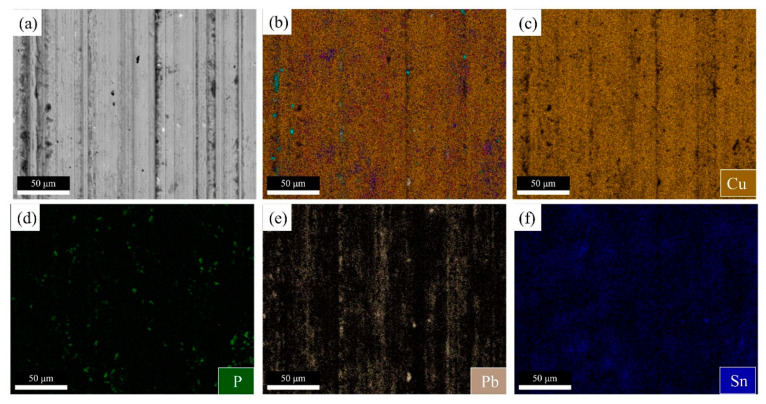
Map of copper layers wear scar with solidification temperature of 700 °C. (**a**) Copper layers BSE, (**b**) element distribution, (**c**) Cu, (**d**) P, (**e**) Pb, and (**f**) Sn.

A surface scan on the wear surface at 700 °C is shown in Figure 10. The Pb film is uniformly distributed on the α-Cu surface, and the Cu_3_P and δ phases are distributed in the wear scar, indicating that it plays a role in reducing wear [26]. When the solidification temperature is 700 °C, there are some liquid islands distributed inside the α-Cu matrix; when these liquid islands solidify and nucleate, the low melting point eutectoid phase (δ + Cu_3_P) is distributed around α-Cu, wrap the α-Cu crystal grains, and part of the eutectoid phase (α + δ + Cu_3_P) into the α-Cu. As the solidification progresses, the remaining eutectoid phase inside the α-Cu is retained, and finally formed in the peritectic structure with Pb coexisting. The Pb film is not easily destroyed during the wear process, which enhances the wear resistance of the copper layer.

## 4. Conclusions

The solid–liquid composite method was used to prepare a steel–copper bimetal sample through the two-stage cooling process (forced air cooling and oil cooling). The aim of the experiments is to identify a solidification temperature that avoids cracking between the steel and copper interface, with a desirable microstructure to optimize friction and wear properties. Solidification temperatures of 400 °C, 500 °C, 600 °C, 700 °C, 800 °C and 900 °C were investigated, and the following conclusions are drawn:

(1) The bimetal copper layer friction and wear parameters are 250 N, speed is 1500 r/min (3.86 m/s), solidification temperature is 400–900 °C, and the friction coefficient is 0.06–0.1, reaching the lowest point of 0.06 at 700 °C;
(2) As the solidification temperature decreases, the growth of primary and secondary dendrites gradually tends to balance at 700 °C, which is lower than this temperature, and the primary dendrite arms gradually grow up. The uniform dendrite distribution makes the Pb distribution uniform, which is beneficial to the friction performance;
(3) The low melting point eutectoid phase (α + δ + Cu_3_P) is distributed around α-Cu, forming a peritectic structure coexisting with Pb. Wearing the process, Pb forms a self-lubricating film uniformly distributed on the surface of α-Cu, and Cu_3_P and δ phases are distributed in the wear mark to increase α-Cu wear resistance.

## Figures and Tables

**Table 1:** Chemical composition of the matrix material (wt.%).

Copper Layer	Pb	Sn	P	Ni	Zn	Cu
EN CC497K	18.35	4.88	0.08	1.83	1.75	Bal.

## Data Availability

Not applicable.
